# Measuring the incidence and prevalence of obstetric fistula: approaches, needs and recommendations

**DOI:** 10.2471/BLT.14.141473

**Published:** 2014-12-01

**Authors:** Özge Tunçalp, Vandana Tripathi, Evelyn Landry, Cynthia K Stanton, Saifuddin Ahmed

**Affiliations:** a Department of Reproductive Health and Research, World Health Organization, avenue Appia 20, 1211 Geneva 27, Switzerland.; b Fistula Care Plus, EngenderHealth, New York, United States of America (USA).; c Department of Population, Family and Reproductive Health, Johns Hopkins Bloomberg School of Public Health, Baltimore, USA.

Obstetric fistula – an abnormal connection between the vagina, rectum and/or bladder – may develop after prolonged and obstructed labour and lead to continuous urinary or faecal incontinence. Most fistulas occur in countries in sub-Saharan Africa or south Asia with poorly-resourced health systems. Women with obstetric fistula are indicators of the failure of health systems to deliver accessible, timely and appropriate intrapartum care. Incidence and prevalence measurements of obstetric fistula are needed to sustain interest in – and funding for – sustainable methods for prevention and treatment. Knowing the absolute numbers of women requiring treatment is also essential for effective health-care planning.

Incidence and prevalence estimates of obstetric fistula are generally based on self-reporting, personal communication with surgeons, studies by advocacy groups and reviews of hospital services in which the relevant denominators are unknown or unreported.^1^ The World Health Organization estimates that between 50 000 to 100 000 women worldwide develop obstetric fistula each year.^2^ However, these estimates are based on scanty data and need to be updated. The relative rarity of obstetric fistula and the geographical remoteness of the areas where most cases occur mean that there are few reliable estimates of the number of women affected. A recent review of 19 studies attempted to estimate the global prevalence and incidence of obstetric fistula but could find very few studies that used a nationally-representative sample or were conducted in South Asia.^3^ Data on the incidence of iatrogenic fistula – a complication of obstetric or gynaecological surgery – are also scarce.

These cross-sectional prevalence studies and prospective and retrospective incidence studies have mostly used community or facility sampling or a combination of both.^3^ Whatever the approach, it is important to report the methods used, the assumptions made and the strengths and limitations of these approaches.

## Facility-based studies

Facility-based studies of prevalence or incidence can be easier to conduct than community-based studies and provide opportunities for clinical assessments and accurate diagnoses. The combination of existing medical records and study-based observations often permits comprehensive data collection. Facility-based studies may be entirely retrospective – if medical records have been kept carefully^4^ – or prospective. However, given the rarity of obstetric fistula, prospective studies may identify very few cases.^5^ Facility-based studies are limited to women who access the facility. Women who do not access facilities are excluded from these studies. These are usually women in remote and rural areas who deliver at home and without a skilled birth attendant – i.e. those most at risk of obstetric fistula. In such studies, the denominator for any estimate is restricted to the study facilities or, at best, their catchment areas.

## Community-based surveys

Compared to facility-based studies, community-based surveys generally provide wider coverage, better representation of a regional or national population and more opportunities to collect a wide range of data – e.g. on the incidence of obstructed labour or stillbirth. However, such surveys can be expensive and time-consuming. Researchers often identify cases of obstetric fistula simply by interviewing women. Some interviewees may report incontinence caused by other conditions while others may be too embarrassed to report incontinence – potentially leading to overestimation and underestimation of the burden of obstetric fistula, respectively. Furthermore, many surveys that address reproductive health issues restrict their samples to women of reproductive age – generally defined as females aged 15–49 years – but obstetric fistula can be found in both younger and older females.^6^

Since 2004, questions about obstetric fistula have been included in the Demographic Health Surveys of more than 20 countries – the majority of them in sub-Saharan Africa. Between 2008 and 2013, 11 of these countries reported the percentages of females aged 15–49 years who, when interviewed in Demographic Health Surveys, claimed that they had heard of obstetric fistula, including those who reported having such a fistula at any time (Table 1). Despite the limitations of self-reporting and a restricted sample due to the age limitations, these observations provide a large multi-country data set for analysis.

**Table 1:** Knowledge and prevalence of obstetric fistula among women, 2008–2013

Country	Year(s)	% of women aged 15–49 years^a^	
		Heard of obstetric fistula	Had obstetric fistula^b^
Burkina Faso	2010	30.9	0.1
Cameroon	2011	23.2	0.4
Congo	2011–2012	17.4	0.3
Guinea	2012	41.8	0.6
Kenya	2008–2009	NA	0.9
Malawi	2010	NA	0.6
Niger	2012	44.1	0.2
Nigeria	2008	30.7	0.4
Senegal	2010–2011	22.2	0.1
Uganda	2011	NA	2.0
United Republic of Tanzania	2010	67	0.5

NA: not available.

^a^ The data were recorded during Demographic Health Surveys that included questions related to obstetric fistula.

^b^ A woman was considered to have had an obstetric fistula if she answered yes to the question “Have you ever experienced a constant leakage of urine or stool from your vagina during the day and night?”

Data source: DHS Program Office.^7^

## Combination studies

Attempts have been made recently to combine community and facility-based studies. Researchers arrange for women with suspected obstetric fistula that they find through a community-based survey to be examined and treated at a health facility. As a method of estimating the incidence or prevalence of obstetric fistula, this can be faster, cheaper and more accurate than a Demographic Health Survey.

One study used a key-informant method to estimate the prevalence of obstetric fistula in the area of Wau – the second largest city in South Sudan.^8^ Using pre-existing networks, the researchers recruited and trained individuals who could act as key informants. After training, the informants returned to their communities and identified a total of 10 potential cases of obstetric fistula among women aged 15–49 years. All 10 cases were then confirmed by an obstetrician in a mobile clinic.

Another study attempted to quantify the numbers of untreated females in two Nigerian states while assessing the usefulness of questions that had been included in the Nigerian Demographic Health Survey.^9^ Four weeks before starting screening, researchers implemented outreach efforts; advocacy with traditional and religious leaders, village heads, government staff and health educators. Trained nurse-midwives conducted screening in health facilities close to where women lived, and referred women with signs of obstetric fistula to higher-level health facilities for treatment. Lessons learnt from this study include the importance of ensuring: (i) community participation and ownership; (ii) context-specific messaging; and (iii) transport for women to the health facility. Researchers also established the fact that mid-level providers can identify fistula cases.

A study in Pakistan applied a mixed community and facility approach to estimate prevalence.^10^ A household survey identified 581 females aged at least 15 years who had incontinence, including 24 who reportedly had symptoms of an obstetric or iatrogenic vaginal fistula. All 24 suspected cases were confirmed – as cases of obstetric (*n* = 20) or iatrogenic (*n* = 4) fistula – by clinical examination.

## Model-based estimation

Data collected in facility- or community-based or combination studies can be used in – and be augmented by – mathematical models that apply probabilistic techniques. Although various models have been used, the difficulty of estimating the prevalence or incidence of very rare events persists.^3^^,^^11^ Methods need to be critically reviewed and strengthened, and approaches that combine facility- and community-based studies need to be further developed. While estimates can help in planning prevention and treatment programmes, these cannot compensate for the lack of good quality data.

## Next steps

Given the rarity of obstetric fistula, there is no single solution to address the problem of the accurate measurement of the condition’s prevalence and incidence. However, there are several steps that can be taken to generate better data for planning. For example, the sporadic information on obstetric fistula gathered from individual studies needs to be replaced or supplemented with data collected through routine surveillance and monitoring that is integrated in health systems and national programmes. A promising development is the incorporation of core indicators related to obstetric fistula in the national health management information systems of several countries (Table 2).

**Table 2:** National indicators for the treatment of obstetric fistula, 2013

Indicator (number of)	Included in national HMIS					
	Bangladesh	Guinea	Mali	Niger	Nigeria	Uganda
**Screening and treatment**						
Women referred for fistula	No	No	No	No	Yes	No
Women presenting with incontinence	No	No	Yes	No	Yes	No
Women referred for incontinence	No	No	Yes	No	No	No
Fistula cases diagnosed	No	Yes^a^	Yes	Yes^b^	No	Yes
Women receiving a fistula repair	No	No	No	Yes	Yes	Yes
Fistulas repaired	Yes	Yes	Yes	Yes	No	No
Women treated by catheter for fistula	No	No	No	Yes	No	No
Cases of vesicovaginal fistula	No	No	No	Yes	No	No
Cases of rectovaginal fistula	No	No	No	Yes	No	No
New cases of fistula	No	No	No	Yes	Yes	No
Women receiving a second repair	No	No	No	No	Yes	No
Women discharged	No	No	No	No	Yes	No
Fistulas closed and dry	Yes	Yes	Yes	Yes	Yes	Yes
Women receiving repair who were discharged as “not closed”	Yes	No	No	No	No	Yes^c^
Women receiving repair who remained incontinent at discharge	Yes	No	No	Yes	No	No
**Capacity to treat**						
Staff capable of fistula surgery	No	No	Yes	No	No	No
Staff capable of clinically diagnosing fistula	No	No	Yes	No	No	No
**Reintegration**						
Women benefitting from a social reintegration programme	No	No	No	Yes	No	No

HMIS: Health Management Information System.

^a^ Number of women with fistula registered.

^b^ Number of women needing fistula repair.

^c^ Number of women receiving repair who were discharged “not closed” or “closed with some remaining incontinence”.

Data source: EngenderHealth/Fistula Care.^12^

At the population level, countries that are conducting Demographic Health Surveys and where home delivery is common should consider including questions relating to obstetric fistula in their survey. A diagnostic algorithm based on the answers to such questions should be developed and validated in multiple settings. Several recent Demographic Health Surveys – e.g. the 2011 survey in Ethiopia and the 2013 survey in Nigeria – included no fistula-related questions and therefore missed opportunities for generating prevalence estimates. Demographic surveillance sites may also generate data for epidemiological research on obstetric fistula.^13^ Further research – ideally integrated within national programmes – should apply mixed community- and facility-based approaches to estimate the numbers of women needing surgery and refer such women to clinical care.

Continuous surveillance of women seeking care is necessary to track the met need for surgical repair at subregional, regional and national levels. In addition to stand-alone fistula centres in countries where obstetric fistula is relatively common, maternity-care facilities should offer diagnosis and surgical repairs to be able to provide a continuum of care. Other approaches focusing on care providers include training them to diagnose fistula during postpartum visits, to recognize iatrogenic fistula, and surveying them on their referral practice for women with fistula-like symptoms. Focus should also be made on outreach and prevention services for rural, malnourished women, since they are at greatest risk.

The burden posed by obstetric fistula – and the resources needed to address it – will only be accurately assessed when there is regular collection of relevant data of good quality at community, facility and country level.
